# *In Vitro* Anthelmintic Activity of Crude Extracts of *Artemisia herba-alba* and *Punica granatum* against *Haemonchus contortus*

**DOI:** 10.1155/2020/4950196

**Published:** 2020-01-27

**Authors:** Aliyi Hassen Ahmed, Mebrat Ejo, Teka Feyera, Dereje Regassa, Bahar Mummed, Solomon Assefa Huluka

**Affiliations:** ^1^Department of Biomedical Sciences, College of Veterinary Medicine and Animal Science, University of Gondar, Ethiopia; ^2^Animal Science, School of Environmental and Rural Science, University of New England, Armidale, NSW 2351, Australia; ^3^College of Veterinary Medicine, Haramaya University, Ethiopia; ^4^Department of Pharmacology and Clinical Pharmacy, School of Pharmacy, Addis Ababa University, Ethiopia

## Abstract

Gastrointestinal nematodes (GINs) are the major limiting factor for the successfulness of livestock production throughout the world. Emergence of resistance strains as well as scarcity and high cost of the currently available drugs has led to the evaluation of other alternative helminth control options, mainly from plants. The current study is aimed at investigating the *in vitro* anthelmintic efficacy of crude methanolic extracts of two traditionally important medicinal plants, *Artemisia herba-alba* and *Punica granatum*, against *Haemonchus contortus* using adult motility assay (AMA) and egg hatch inhibition assay (EHIA). Four graded concentrations of the extracts were tested for both the AMA (10, 5, 2.5, and 1.25 mg/mg) and EHIA (0.1, 0.25, 0.5, and 1 mg/mL) in replicates. Albendazole and phosphate-buffered saline (AMA) or distilled water (EHIA) were used as the positive and negative controls, respectively. The crude extracts of *A*. *herba-alba* and *P*. *granatum* exhibited a potential anthelmintic activity at all dose levels in a concentration- and time-dependent fashion. The highest concentration (10 mg/mL) of all the extracts caused a significantly (*p* < 0.05) superior nematocidal activity compared to the negative control. Moreover, significant and concentration-dependent egg hatching inhibition effect was observed from both plant extracts. Maximal (98.67%) egg hatching inhibition effect was exhibited by the flower extract of *A*. *herba-alba* at 1 mg/mL concentration. The relative egg hatch inhibition efficacy indicated that both plants caused a significantly (*p* < 0.05) greater egg hatch inhibition within 48 hr of exposure. The current study validated the traditional use of both plants as a natural anthelmintic against *H*. *contortus* justifying a need to undertake detail pharmacological and toxicological investigation on both plants.

## 1. Introduction

Gastrointestinal nematodes (GINs) remain a major threat to the health and welfare of small ruminants throughout the world [1]. GINs represent a major economic hurdle in ruminant systems through mortality, weight loss, and reduced milk and meat production [2, 3]. In Ethiopia, a total loss of US $81.8 million is reported annually due to helminth parasites [4].

*Haemonchus contortus* is an important abomasal helminth of small ruminants responsible for disease and major production losses worldwide [5]. Moreover, it is one of the major livestock parasites in tropical and temperate farming areas [6]. Compared to other nematodes, *H*. *contortus* is a highly pathogenic parasite of small ruminants and is capable of causing acute disease and high mortality in all classes of stock [7]. Heavy burdens of this blood-feeding parasite can cause severe anemia and rapid death in affected livestock [5]. It was reported to be one of the top ten constraints of sheep and goat production in East Africa [7].

GIN control has an important role to play in improving livestock production from a limited natural resource base and to improve animal health and welfare [8]. Synthetic anthelmintic agents are commonly employed to control GINs [9]. However, inappropriate and exclusive application of these drugs has contributed to the development of extensively drug-resistant parasites. This, in turn, increased risks of residues in the meat and milk of these animals and the environment [5, 10]. Additionally, these synthetic agents are likely unaffordable and expensive. Consequently, demand for alternative control measures has constantly increased during the last years [1]. Hence, medicinal plants practiced in folk medicine can serve as a source of affordable and effective anthelmintic agents [11]. In the current study, we have attempted to correlate traditionally claimed anthelmintic activity of two plants in the context of experimental evidences.

The plant *Artemisia herba-alba* (Chukun in Amharic), which belongs to the Asteraceae family, is used as an anthelmintic agent as well as for other common applications in folk medicine [12, 13]. From the Lythraceae family, *Punica granatum* (Roman) is used against GI nematodes [14]. *P*. *granatum* has been used in natural and holistic medicine to expel tapeworms and treat various ailments [15, 16]. The aim of the present study was thus to evaluate the in vitro anthelmintic effect of extracts from these plants against *H*. *contortus*.

## 2. Materials and Methods

### 2.1. Collection of Plant Samples

Based on a preliminary interview conducted among livestock raisers of Midaga-Tola district, two plants (*A*. *herba-alba* and *P*. *granatum*) were selected. These plants were commonly used as anthelmintic agents in Ethiopian folkloric medicine. Therefore, fresh flower and aerial parts (stem and leaves) of *A*. *herba-alba* and peel and root parts of *P*. *granatum* were collected from their natural habitat around Midaga-Tola district, East Hararghe zone, 582 km away from Addis Ababa. Herbal identification of the collected plants was then made by a taxonomist at the herbarium of Plant Science Department, Haramaya University, where a voucher specimen (AH001/17 for *A*. *herba-alba* and AH002/17 *P*. *granatum*) were deposited for future references.

### 2.2. Plant Extract Preparation

The collected plant materials were cleaned, shade dried, mechanically ground, and coarsely powdered using a laboratory mortar and pestle. Then, the powdered specimens were subjected to a cold maceration extraction technique using the methanol solvent system for 72 hr. For each sample, a total of 250 g of the coarsely powdered plant materials was separately soaked in the extraction solvent (1 : 10). The extraction process was facilitated using a mechanical shaker at 120 rpm. The same volume of solvent was used to remacerate the residue for another 72 hr, twice. Finally, the filtrates were recombined and concentrated on rotavapor (Buchi, Switzerland) at 40°C under reduced pressure. Moreover, the concentrated filtrate was freeze-dried in a lyophilizer to earn a dried extract.

The dried extract was weighed and provided a percent yield of 14.3% (*w*/*w*) and 12.5% (*w*/*w*) for the flower and aerial parts of *A*. *herba-alba*, respectively. Extract from the peel and root of *P*. *granatum*, on the other hand, yielded 13% (*w*/*w*) and 9.4% (*w*/*w*), respectively. The resulting extracts were transferred into well-labeled vials and kept in a refrigerator until required for use.

### 2.3. Phytochemical Screening

All extracts were screened for the presence and absence of different phytochemicals. Standard screening tests using conventional protocol, procedure, and reagents were conducted to identify the constituents as described in Trease and Evans [17] and Sofowora [18].

### 2.4. Biological Assay

#### 2.4.1. Collection of Parasites

Adult parasites of *H*. *Contortus* were collected from the abomasum of a sheep obtained from Haramaya municipal abattoir. The abomasum was collected immediately after slaughtering and transported to Veterinary Parasitology laboratory of Haramaya University. In the laboratory, abomasum was washed by running water and worms were then isolated by incising the greater curvature of the abomasa and the parasites were kept in phosphate buffer saline (PBS) until the *in vitro* evaluation was started. The female worms were then ground using a mortar and pestle to liberate the eggs.

#### 2.4.2. Adult Motility Assay (AMA)

A total of about 368 adult *H*. *contortus* parasites were used to assess the anthelmintic effect of extracts against mature *H*. *contortus* worms on adult motility assay (AMA), according to the technique described by Sharma et al. [19]. Each plant extract was tested on different concentrations (10, 5, 2.5, and 1.25 mg/mL) prepared in PBS.

The assay was conducted in six groups. Group I and Group II received crude methanol extract from the aerial and flower parts of *A. herba-alba*, respectively. Group III was treated with crude methanol extract of *P*. *granatum* peel part while Group IV received the methanol extract of *P*. *granatum* root part. Group V and VI received 0.25 mg/mL of albendazole (positive control) and PBS (negative control), respectively.

Inhibition of motility was taken as an indication of worm mortality/paralysis. To assess the motility inhibition effect of the extracts, the observations were taken at regular time intervals until the 7^th^ hour after treatment. Worms not showing any motility were taken out and placed in lukewarm PBS for 10 minutes and, in case of revival in motility, the observed worms were counted as alive; otherwise, they were counted as dead.

#### 2.4.3. Egg Hatch Inhibition Assay (EHIA)

The ability of the extracts to inhibit egg hatching was conducted according to the procedure described by Coles et al. [20]. Eggs were washed thrice with distilled water and adjusted to a concentration of 100-200 eggs/mL using the McMaster technique [21]. The suspension was centrifuged for 5 minutes at 1500 rpm and the supernatant was discarded. Approximately, 100 eggs in 200 *μ*L of distilled water were pipetted into each well of a 48-well microtiter plate. To each of the test wells, 200 *μ*L of each plant extract at concentrations of 0.1, 0.25, 0.5, and 1 mg/mL was added to a final volume of 400 *μ*L per well. Similarly, 200 *μ*L of albendazole (99.8% pure standard reference) at a concentration of 0.25 mg/mL was used as a positive control, while distilled water (200 *μ*L) was used as a negative control. The experiment was conducted in duplicates for each concentration and replicated three times. In this assay, all plates were incubated at 37°C for 48 hr. A drop of Lugol's iodine solution was added to each well to stop further hatching, and all the unhatched eggs and L1 larvae in each well were counted under a dissecting microscope. Finally, percent inhibition of egg hatching was calculated:

Percent inhibition = 100(1 − *P*_test_/*P*_control_), where *P* = number of eggs hatched in EHIA.

#### 2.4.4. Data Analysis

Data were organized, edited, and analyzed using SPSS Version 20. Results generated from both assays were analyzed with one-way ANOVA followed by Tukey's HSD multiple comparison. *p* value of less than 0.05 was considered statistically significant.

## 3. Results

### 3.1. Phytochemical Screening

The preliminary phytochemical screening of the plant materials revealed the presence of alkaloids, saponins, flavonoids, tannins, glycosides, and phenols in all of the tested extracts (Table 1). Moreover, the strong presence of alkaloids, tannins, flavonoids, glycosides, and phenols were detected from the root crude extract of *P*. *granatum*.

### 3.2. Adult Motility Test

**Figure 1:**
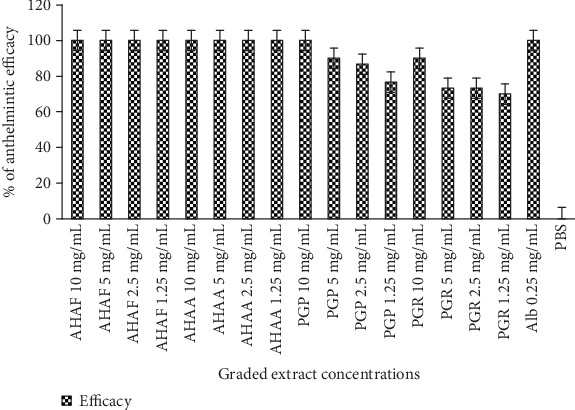
Relative nematocidal efficacy of graded concentration of crude extracts of *A*. *herba-alba* and *P*. *granatum*. AHAF: *A*. *herba-alba* flower part, AHAA: *A*. *herba-alba* aerial part, PGP: *P. granatum* peel part, PGR*: P*. *granatum* root part, Alb: albendazole, PBS: phosphate buffer saline.

The present study indicated that all concentrations of methanolic flower and aerial part extracts of *A*. *herba-alba* as well as the highest concentration of methanolic peel extract of *P*. *granatum* produced a relatively comparable anthelmintic activity with the conventional anthelmintic agent, albendazole (Figure 1). The anthelmintic activity of plant extracts increased with time. Accordingly, after 7 hr exposure of adult *H*. *contortus* to the highest concentration (10 mg/mL) of extracts, both plants produced a significant (*p* < 0.05) mortality of adult *H*. *contortus*. Albendazole, on the other hand, killed all parasites within 5 hr at a concentration of 0.25 mg/mL (Table 2).

### 3.3. Egg Hatching Inhibition Assay

**Figure 2:**
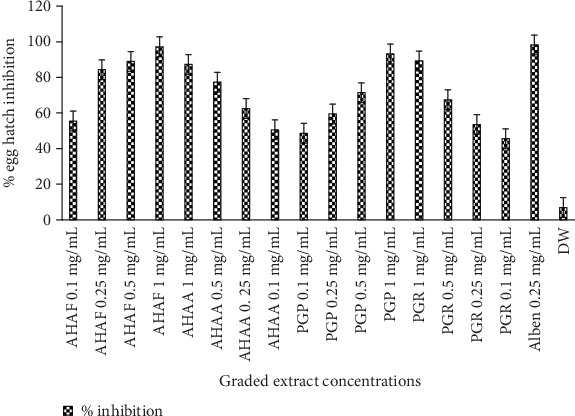
Relative egg hatching inhibition efficacy of graded concentration of crude extracts of *A*. *herba-alba* and *P*. *granatum*. AHAF: *A*. *herba-alba* flower part, AHAA: *A*. *herba-alba* aerial part, PGP: *P*. *granatum* peel part, PGR: *P*. *granatum* root part, Alben: albendazole, DW: distilled water.

Both *A*. *herba-alba* (flower and aerial extract) and *P. granatum* (peel and root extract) induced a significant egg hatching inhibition effect in a concentration-dependent manner. Flower and aerial part methanolic extract of *A. herba-alba* exhibited a 98.67% and 88.3% inhibition, respectively, at 1 mg/mL concentration. Furthermore, the egg hatch inhibitory efficacy profile of *P*. *granatum* extracts, as percentage of eggs unhatched at the end of the observation period, is as follows; 49.33 and 46.33% at concentration 0.1 mg/mL, 60.67 and 54.33 at 0.25 mg/mL, 72.67 and 68.33 at 0.5 mg/L, and 94.63 and 90.33 at 1 mg/mL concentration of peel and root crude extracts, respectively (Figure 2).

Values are mean ± SEM. All superscripts indicate significance at *p* < 0.05,^a^compared to untreated (PBS), ^b^compared to albendazole, and ^c^compared to the lowest concentration of methanolic extract of *A*. *herba-alba* flower, and ^d^compared to the lowest concentration of methanolic extracts of *A*. *herba-alba* aerial part, ^e^compared to the lowest concentration of methanolic extract of *P*. *granatum* peel part, and ^f^compared to the lowest concentration of methanolic extract of *P. granatum* root.

## 4. Discussion

The emergence of resistant strains, the presence of anthelmintic drug residues in animal products, and synthetic drugs' toxicity have led to a rebirth of interest in the use of natural products [22]. Plant materials tested for their *in vitro* anthelmintic activity in the present study have been identified by local livestock raisers. *In vitro* techniques such as the AMA and EHIA are preferred to *in vivo* methods due to their low cost, simplicity, and rapid turnover [23]. Moreover, for *in vitro* studies, *H*. *contortus* is proved to be a good test worm because of its longer survival in PBS. This abomasal helminth has recently been used for *in vitro* studies by other workers [23, 24].

In the current *in vitro* study, 10 mg/mL concentration of methanol peel extract of *P*. *granatum* produced a statistically significant anthelmintic activity that is comparable with the conventional anthelmintic agent, albendazole. This finding is additionally in line with the clinical study that confirmed the efficacy of the plant against nematodes in calves [25] and superior to an *in vitro* study that reported a moderate level of anthelmintic activity from the rind of *P*. *granatum* [26]. Moreover, similar to a study done by Prakash et al. [27] on the alcoholic extract of *P*. *granatum*, our study showed a significant anthelmintic activity of the plant as revealed by a concentration-dependent inhibition of transformation of eggs to the larva of *H*. *contortus.*

Some previous works similarly indicated that *P*. *granatum* has a marked effect on cestode and nematodes [28] as well as protozoan infections [29]. Moreover, our study substantiated a previous report on the traditional application of *P*. *granatum* plant, in which various parts of the plant can be used as a traditional anthelmintic agent [30, 31].

The plant, *A*. *herba-alba*, is mainly used as an anthelmintic agent in traditional practice [10]. Concordant with this, in EHIA of the present study, flower part methanol extract of *A*. *herba-alba* induced a significant egg hatching inhibition of 98.67%, at 1 mg/mL concentration. This is in line with a study done by Boonmasawai et al. [11] in which shoot parts are used as an anthelmintic in *H*. *contortus* infestation of sheep as the result of its santonin. The result exhibited by the plant in AMA, moreover, is in agreement with a previously reported activity of a plant in the same genus, *A*. *absinthium*, which significantly affected motility and viability of *H*. *contortus*, *in vitro* [32]. Furthermore, the genus is a rich source of sesquiterpene lactones and flavonoids that might have anthelmintic activity with low risk of mammalian toxicity [33].

The exhibited anthelmintic effect of the two plants might be attributed to the existing secondary metabolites. Joshi et al. [34] assimilated that tannins may exert anthelmintic activity by reducing hatching, blocking its development to the infective larval stage and decrease in adults' motility. Besides, tannins have been shown to interfere with coupled oxidative phosphorylation and block ATP synthesis in *H*. *contortus* [35]. Wang et al. [36] has confirmed the anthelmintic efficacy of plant-based alkaloids. The environmental stimuli on the host lead to the release of enzymes by larvae, which degrade the egg membrane [37]. The action of alkaloids in these two plants might be linked to the inhibition of these enzymes' activity.

Once a plant has proven its efficiency *in vitro*, further *in vivo* testing will be necessary to confirm the obtained results and evaluate risks, side effects, and future applicability [38]. Therefore, *in vivo* anthelmintic evaluation of these plants is imperative prior to their clinical use.

## 5. Conclusion

The *in vitro* anthelmintic activity of tested plants is characterized by a decrease in hatching and reduced motility of the larvae and adult stage of *H*. *contortus*. Accordingly, they have the potential to contribute in controlling gastrointestinal parasites of ruminants. Therefore, fractionation of the crude extracts and isolation of compounds to further evaluate the anthelmintic efficacy of these plants involving other parasite developmental stages are warranted.

## Figures and Tables

**Table 1:** Phytochemical constituents of investigated plant extracts.

Extracts	Phytochemicals						
	Alkaloid	Saponin	Tannins	Flavonoids	Glycosides	Sterols	Phenol
*A*. *herba-alba*							
Flower part	+++	++	+++	++	+	-	+++
Aerial part	++	++	+	++	++	-	++
*P*. *granatum*							
Peel part	++	++	++	++	+++	-	++
Root part	+++	+	+++	+++	+++	-	+++

Key: +++ = strongly detected, ++ = moderately detected, + = slightly detected, and - = absent.

**Table 2:** *In vitro* nematocidal effect of crude extracts of different parts of *A*. *herba-alba* and *P*. *granatum* against *H*. *contortus*.

Treatment	Concentration (mg/mL)	1 hr	2 hr	3 hr	4 hr	5 hr	6 hr	7 hr
*A*. *herba alba* flower	10	4.00 ± 0.58^cdef^	5.00 ± 0.88^defa^	6.33 ± 0.88^efa^	7.67 ± 0.67^efa^	8.67 ± 0.67^efa^	9.33 ± 0.33^efa^	10.00 ± 0.00^efa^
	5	3.00 ± 0.58	4.67 ± 0.33	6.00 ± 0.33	7.33 ± 0.67	8.33 ± 0.33	9.33 ± 0.33	10.00 ± 0.00
	2.5	2.33 ± 0.33	3.67 ± 0.33	5.33 ± 0.33	6.67 ± 0.33	8.00 ± 0.00	9.67 ± 0.33	10.00 ± 0.00
	1.25	1.33 ± 0.33^ceb^	3.33 ± 0.33^a^	5.33 ± 0.33^efba^	6.67 ± 0.33^fba^	8.33 ± 0.33^efa^	9.67 ± 0.33^efa^	10.00 ± 0.00^efa^

*A*. *herba alba* aerial part	10	4.00 ± 0.58^cdefa^	5.67 ± 0.33^defa^	6.67 ± 0.33	7.67 ± 0.33^efa^	8.67 ± 0.33^efa^	9.67 ± 0.33^efa^	10.00 ± 0.00
	5	2.00 ± 0.58	3.33 ± 0.33	4.67 ± 0.33	6.00 ± 0.58	7.67 ± 0.33	9.00 ± 0.00	10.00 ± 0.00
	2.5	1.67 ± 0.33	3.33 ± 0.33	4.67 ± 0.33	6.33 ± 0.67	8.33 ± 0.33	9.33 ± 0.33	10.00 ± 0.00
	1.25	1.33 ± 0.33^cdeb^	2.67 ± 0.33^cdeba^	4.00 ± 0.58^ba^	5.00 ± 0.58^ba^	7.00 ± 0.58^ba^	8.67 ± 0.33^abcd^	10.00 ± 0.00^efa^

*P*. *granatum* peel part	10	4.33 ± 0.33^cdefa^	5.67 ± 0.33^defa^	6.67 ± 0.33^efa^	7.67 ± 0.33^efa^	8.67 ± 0.33^efa^	9.67 ± 0.33^efa^	10.00 ± 0.00^efa^
	5	2.00 ± 0.58	3.00 ± 0.58	4.00 ± 0.58	5.00 ± 0.58	6.00 ± 0.58	7.67 ± 0.33	9.00 ± .58
	2.5	1.33 ± 0.33	2.00 ± 0.58	3.67 ± 0.88	4.67 ± 0.9	6.00 ± 0.58	7.33 ± 0.33	8.67 ± 0.33
	1.25	0.33 ± 0.33^cdefb^	1.00 ± 0.58^cdefb^	2.00 ± 0.58^cdeb^	4.00 ± 0.58^cdeba^	5.00 ± 0.58^cdba^	6.67 ± 0.33^cdba^	7.67 ± 0.33^cdeba^

*P*. *granatum* root part	10	3.00 ± 0.58^cdefa^	4.00 ± 0.58^efa^	5.00 ± 0.58^efba^	6.00 ± 0.58^ba^	7.00 ± 0.58^ba^	8.00 ± 0.58^fba^	9.00 ± 0.58^fa^
	5	1.00 ± 0.58	2.00 ± 0.58	3.00 ± 0.58	4.33 ± 0.67	5.33 ± 0.67	6.33 ± 0.67	7.33 ± 0.67
	2.5	0.33 ± 0.33	1.33 ± 0.33	2.67 ± 0.33	3.67 ± 0.33	5.00 ± 0.00	6.33 ± 0.33	7.33 ± 0.33
	1.25	0.00 ± 0.00^cdefb^	1.00 ± 0.58^cdefb^	2.00 ± 0.58^cdefb^	3.67 ± 0.68^cdeba^	4.67 ± 0.68^cdeba^	6.00 ± 0.58^cdefa^	7.00 ± 0.58^cdefba^

Albendazole	0.25	4.00 ± 0.58^cdefa^	5.67 ± 0.33^defa^	8.33 ± 0.33^cdefb^	9.67 ± 0.00^cdefa^	10.00 ± 0.00^defa^	10.00 ± 0.00^efa^	10.00 ± 0.00^efa^

PBS	0.00 ± 0.00	0.00 ± 0.00^cdefb^	0.00 ± 0.00^cdfb^	0.00 ± 0.00^cdefb^	0.00 ± 0.00^cdefb^	0.00 ± 0.00^cdefb^	0.00 ± 0.00^cdeb^	0.00 ± 0.00^cdeb^

## Data Availability

Upon reasonable request, the supporting dataset are available from the corresponding author. Moreover, the dried specimen of tested plants and their voucher numbers are deposited in the herbarium of Haramaya University.

## Abbreviations

AMA: Adult motility assay
ATP: Adenosine triphosphate
EHIA: Egg hatch inhibition assay
GINs: Gastrointestinal nematodes
PBS: Phosphate buffer saline.
